# Advanced deep learning techniques for early disease prediction in cauliflower plants

**DOI:** 10.1038/s41598-023-45403-w

**Published:** 2023-10-27

**Authors:** G. Prabu Kanna, S. J. K. Jagadeesh Kumar, Yogesh Kumar, Ankur Changela, Marcin Woźniak, Jana Shafi, Muhammad Fazal Ijaz

**Affiliations:** 1 School of Computer Science and Engineering, VIT Bhopal University, Bhopal-Indore Highway, Kothrikalan, Sehore Madhya Pradesh - 466114, India; 2 Department of Computer Science and Engineering, Kathir College of Engineering, Neelambur, India; 3 Department of CSE, School of Technology, Pandit Deendayal Energy University, Gandhinagar, Gujarat India; 4 Faculty of Applied Mathematics, Silesian University of Technology, 44-100 Gliwice, Poland; 5 Department of Computer Science, College of Arts and Science, Prince Sattam bin Abdul Aziz University, 11991 Wadi Ad-Dawasir, Saudi Arabia; 6 School of IT and Engineering, Melbourne Institute of Technology, Melbourne, 3000 Australia; 7 Department of ICT, School of Technology, Pandit Deendayal Energy University, Gandhinagar, Gujarat, India

**Keywords:** Mathematics and computing, Computer science

## Abstract

Agriculture plays a pivotal role in the economies of developing countries by providing livelihoods, sustenance, and employment opportunities in rural areas. However, crop diseases pose a significant threat to both farmers’ incomes and food security. Furthermore, these diseases also show adverse effects on human health by causing various illnesses. Till date, only a limited number of studies have been conducted to identify and classify diseased cauliflower plants but they also face certain challenges such as insufficient disease surveillance mechanisms, the lack of comprehensive datasets that are properly labelled as well as are of high quality, and the considerable computational resources that are necessary for conducting thorough analysis. In view of the aforementioned challenges, the primary objective of this manuscript is to tackle these significant concerns and enhance understanding regarding the significance of cauliflower disease identification and detection in rural agriculture through the use of advanced deep transfer learning techniques. The work is conducted on the four classes of cauliflower diseases i.e. Bacterial spot rot, Black rot, Downy Mildew, and No disease which are taken from VegNet dataset. Ten deep transfer learning models such as EfficientNetB0, Xception, EfficientNetB1, MobileNetV2, EfficientNetB2, DenseNet201, EfficientNetB3, InceptionResNetV2, EfficientNetB4, and ResNet152V2, are trained and examined on the basis of root mean square error, recall, precision, F1-score, accuracy, and loss. Remarkably, EfficientNetB1 achieved the highest validation accuracy (99.90%), lowest loss (0.16), and root mean square error (0.40) during experimentation. It has been observed that our research highlights the critical role of advanced CNN models in automating cauliflower disease detection and classification and such models can lead to robust applications for cauliflower disease management in agriculture, ultimately benefiting both farmers and consumers.

## Introduction

**Figure 1:**
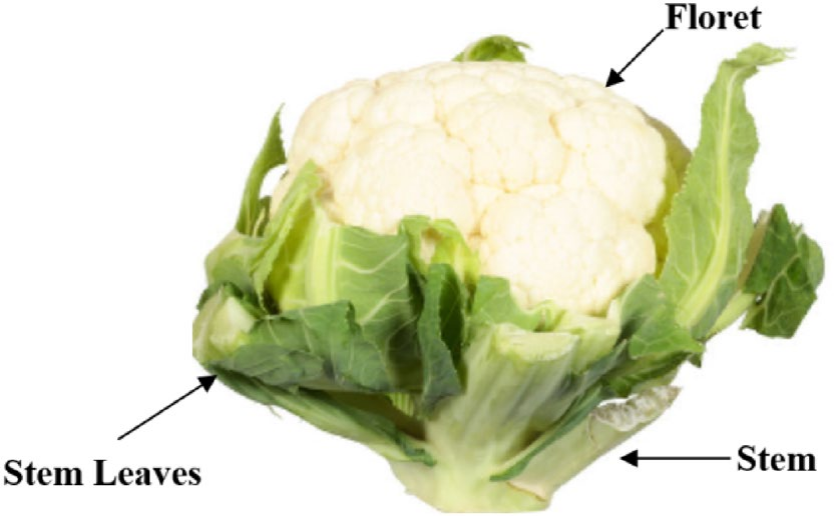
Parts of cauliflower^2^.

“Cauliflower” word has been originated from the Italic word *cavolfiore*, which means “cabbage flower.” It is plant that grows annually and is produced by seed. It comes from *Brassica oleracea*, with the genus *Brassica* and the family Brassicaceae (or mustard). After cabbage, it is the second most popular ‘cole’ crop in the world^1^. The cauliflower consists of parts such as the Head, floret, and stem. The edible part of the cauliflower is head which is also called curd, as shown in Fig. 1. Cauliflower also benefits human health as it contains phytonutrients that reduce cancer risk^2^. In addition, this cruciferous vegetable provides fibre that lowers the chance of heart problems and contains essential nutrients such as choline to help people with good sleep, learning, muscular movement, and sharp memory^3^.

Cauliflowers are grown in various countries across the globe, like India, China, Spain, The USA, Mexico, and Bangladesh, with hundreds of varieties that are being commercialized, as shown in Table 1. As far as India is mentioned, the annual production and acreage of cauliflower are about 7,887,000 million tons and 2.5 lac hectares, respectively^4^.

**Table 1:** Varieties of cauliflower^2^.

Variety	Image	Description
White Cauliflower		It is a popular variety of cauliflower with a white head and green leaves around it
Orange Cauliflower		This type of cauliflower has an orange pigment called beta-carotene and is found in mostly Canada
Green Cauliflower		This type of cauliflower is also as Broccoflower or Broccoli. It is mostly found in the U.S. and Europe
Purple Cauliflower		This cauliflower has anthocyanins that cause purple color to the vegetable. It is found in Britain, Italy

Although cauliflower has various health-based benefits, its disease infection is the main drawback of its production^5^. When cauliflowers are cultivated, they are infected by either bacteria or fungus, which gives birth to various diseases such as bacterial soft rot (Erwinia and Pseudomonas species), blackleg (Leptosphaeria maculans), black rot, downy mildew (Hyaloperonospora parasitica), powdery mildew (Erysiphe cruciferarum), ring spot (*Mycosphaerella brassicicola*), white rust (Albugo Candida), etc.^6^.

The rotten or infected cauliflowers throw a terrible impact on the health of human beings. When they consume them, they cause allergies like sneezing, itching, watery eyes, coughing, difficulty breathing, ear and skin infections, gastrointestinal diseases, etc. The pesticides or insecticides sprayed on them to keep the cauliflowers away from bacteria also cause severe health issues to humans like dizziness, diarrhea, nausea, acute as well as chronic poisoning, Alzheimer, cancer, asthma, bronchitis, etc. Other than human health issues, cauliflower production quality and quantity have also been degraded in the agricultural sector.

In fact, traditional cauliflower disease detection methods suffer from numerous limitations in agriculture. They often rely on subjective human visual inspection, leading to errors and inconsistency. Manual inspection is time-consuming and delays disease detection, enabling rapid infection spread. The cost of training and maintaining agricultural experts for disease identification is prohibitive for many farmers, particularly in remote areas. These methods often miss early or asymptomatic infections, depend on specific environmental conditions, lack data documentation, and are not easily scalable. They rely heavily on expert knowledge, limiting their applicability. In contrast, advanced deep transfer learning techniques offer automated, accurate, fast, and scalable disease detection with continuous crop monitoring, addressing these shortcomings. Hence it is essential to have early detection of such diseases so that appropriate measures will be taken to escalate the profit and yield of cauliflower cultivation^7^.

Artificial intelligence (AI) has become a transformative force across various industries, and its impact on agriculture, a profession employing approximately 58% of India's population, is undeniable^8^. As the population continues to grow exponentially, the challenges in ensuring food security and sustaining agricultural businesses have intensified. Integrating AI into agriculture is pivotal, not only for enhancing agricultural efficiency but also for mitigating adverse environmental impacts. It is imperative that rural farmers, the backbone of the agricultural sector, equip themselves with tools to swiftly detect and address crop-related issues.

Deep learning models have emerged as powerful tools in plant disease detection, offering a potent solution to the challenges faced by the agricultural sector in India and worldwide. These models, especially Convolutional Neural Networks (CNNs), are great at recognizing images. This makes them perfect for studying visual data like images of plant leaves, which are often used to diagnose diseases. Deep learning models learn complex patterns and features by being trained on large sets of labelled pictures of healthy and unhealthy plants. This lets those spot even minor signs of disease^9^ as most of the time, standard methods of detection can’t get to this level of detail. Additionally, deep learning models can also be tuned and changed to fit different types of crops and diseases. This makes them useful in a wide range of farming situations. Their real-time processing capabilities allow for rapid disease identification, offering farmers timely insights to take appropriate action. The integration of deep learning models in handheld devices or smartphones can empower rural farmers with accessible and user-friendly tools for on-the-spot disease diagnosis, ultimately contributing to increased crop yields, sustainable agriculture, and food security^10^.

In this context, AI holds tremendous promise for diagnosing and managing plant diseases, identifying pests, addressing malnutrition in crops, and even detecting and managing weed infestations. The ability of AI to offer practical and effective solutions to these challenges is undeniable. To harness this potential, publicly available large datasets have been harnessed to train machine learning and deep learning algorithms, paving the way for streamlined disease detection and classification in farming crops, including fruits, plants, and vegetables^10^.

Against this backdrop, the primary objective of this paper is crystal clear: to identify and detect various diseases afflicting cauliflower crops using advanced deep-transfer learning techniques. By doing so, our research aims to not only protect agricultural yields but, more importantly, to safeguard human health by preventing the consumption of contaminated produce. The following contributions were made to carry out the research:

- The images of four classes like bacterial spot rot, downy mildew, black rot, and no disease of cauliflower disease dataset is initially taken.
- In the next step, collected image data is pre processed by reducing its original size to 224 × 224, and various morphological operations are applied such as erosion and dilation.
- In this phase, images have been visualized graphically to find out the pixel intensity and generate red green and blue histograms to study the pattern of data.
- Further, characteristics of image data such as mean intensity, min/max value, extent, perimeter, area, etc., are calculated and extreme points are generated to obtain the cropped image. Additionally, adaptive thresholding is also applied so that the background and foreground part of the image can be differentiated to enhance the classification accuracy of the model followed by the splitting of train and test dataset..
- After splitting of the dataset, various transfer learning classifiers such as Xception, EfficientNetB0, EfficientNetB1, EfficientNetB2, EfficientNetB3, EfficientNetB4, MobileNetV2, DenseNet201, ResNet152V2, and InceptionResNetV2 are taken and trained with the dataset.
- In the last phase, the performance of all these models have been examined by computing their accuracy and loss as well as generating the confusion matrix to obtain the values of another set of performance metrics.

### Organization of the paper

In the “Introduction” section, we briefly described cauliflower, its diseases, and its influence on human health, as well as how AI can detect such sick crops. Section “Background” describes researchers’ use of AI learning models to detect plant and cauliflower-based diseases. This research is based on a cauliflower disease detection system, so section “Materials and methods” discusses the method to develop such a system, where the results are analyzed and compared to state-of-the-art results in section “Analyzing the results”*. *Section “Conclusion” concludes the article with problems and future scope.

## Background

Researchers have shown an impressive contribution to detecting and classifying various cauliflower diseases to protect humans from harmful diseases.

The researchers in paper^11^, identify the diseases affecting cauliflower plants, to enhance cauliflower production efficiency in Bangladesh’s agricultural sector. K-means clustering was used for image segmentation following preprocessing, and ten relevant features were extracted. For classification, various methods were assessed, with the Random Forest algorithm achieving an overall accuracy of 81.68%. Additionally, Convolutional Neural Networks (CNNs), MobileNetV2, InceptionV3, VGG16, and ResNet50 were employed, with InceptionV3 achieving the highest accuracy of 90.08% among these methods. The researchers in paper^12^ mentioned about the novel dataset which they had created by collecting the cauliflower leaves. The work was conducted on MATLAB and various traditional machine learning techniques were applied which included decision tree, random forest, support vector machine, naïve Bayes, and sequential minimal optimization to detect diseased leaf.

Likewise, in paper^2^ researchers created VegNet dataset which consist of various classes of diseased cauliflower plants such as black rot, downy mildew, and bacterial spot rot. These photographs were meticulously shot between December 20th and January 15th, when cauliflower plants were in full bloom and illnesses were most visible. Their dataset was rigorously organized and will be used to develop and validate machine learning-based automated algorithms for detecting cauliflower illnesses. An analysis into multiple convolutional neural network (CNN) models paired with transfer learning methods was conducted in research article^13^. The major purpose was to classify four cauliflower diseases: bacterial soft rot, black rot, buttoning, and white rust. This study's dataset includes approximately 2500 photos. InceptionV3 outperformed the other CNN models tested, with a test accuracy of 93.93%.

Likewise, the researchers in paper^14^ presented an online expert system to assist cauliflower farmers in identifying and managing diseases affecting their crops. Their system operated by processing images captured using smartphones or handheld devices, classifying them to pinpoint specific cauliflower diseases. The targeted diseases include ‘black rot,’ ‘bacterial soft rot,’ ‘white rust,’ as well as ‘downy mildew.’ To implement it, they used a dataset comprising 776 images. The process involved initial image segmentation using the K-means clustering algorithm to isolate disease-affected regions. Subsequently, two types of features, statistical and co-occurrence, were extracted from these segmented regions. For disease classification, six different algorithms were employed: Kstar, LMT (Logistic Model Tree), BayesNet, BPN (Back Propagation Neural Network), Random Forest, and J48 where the results indicated that the Random Forest classifier outperformed all others, achieving an accuracy rate of approximately 89.00% for cauliflower disease recognition. The researchers in paper^15^ used LeNet image processing and deep learning techniques for the classification of cauliflower samples into four categories: healthy, powdery mildew-infected, black rot-infected, and bacterial soft rot-infected. A carefully curated dataset of 655 color images representing these categories was employed, with 70% of the data allocated for model training. Results indicated the model's remarkable ability to accurately classify healthy cauliflowers, those with black rot, and those affected by powdery mildew, achieving a perfect 100% classification rate. Additionally, it demonstrated a highly impressive 99% accuracy in identifying cauliflower specimens afflicted by bacterial soft rot. In paper^16^, the researchers proposed a convolutional neural network (CNN) with transfer learning for the detection and classification of surface defects in fresh-cut cauliflower, aiming to overcome the inefficiencies of manual detection methods. The dataset comprises 4,790 cauliflower images categorized as diseased, healthy, mildewed, and browning. To optimize the model, the parameters of MobileNet were fine-tuned to enhance the accuracy and training speed. This involved selecting optimal hyper-parameters, adjusting frozen layer counts, and integrating ImageNet parameters with in-house trained ones. Comparisons were made with InceptionV3, NASNetMobile, and VGG19,. Experimental results highlighted the MobileNet model's exceptional performance, achieving a 0.033 loss, 99.27% accuracy, and a 99.24% F1 score on the test set with specific parameter settings. In the research paper denoted as^17^, an expert system was introduced, which synergized agricultural and medical expertise with machine vision. This system analyzed images taken using smartphones or portable devices to categorize plant diseases, offering valuable support to farmers in managing their crop health issues. The primary focus of their investigation revolved around the detection of eggplant diseases, using a transfer learning technique based on convolutional neural networks (CNNs). Several transfer learning models, including DenseNet201, Xception, and ResNet152V2, were utilized in their study. Among these models, DenseNet201 exhibited the highest level of accuracy, achieving an impressive 99.06% accuracy rate in the identification of diseases. The authors in study^18^ introduced a model to identify diseases in the plant leaves using advanced CNN model. Four deep learning models (Inception V3, VGG16, DenseNet201, and ResNet152V2,) were evaluated for their accuracy in detecting plant diseases. The research also involved the development of a web-based application for diagnosing plant diseases from leaf images. The dataset comprised 28,310 photos of leaves from three crops: potato, pepper, and tomato were taken. Their proposed model achieved impressive results, with a training and validation accuracy of 99.44% and 98.70% respectively in the experiments. In a certain research study referred to as^19^, a novel approach was proposed by the researchers. They combined the capabilities of MobileNetV2 and Xception models by integrating the features they extract, with the goal of improving the performance of plant disease detection. The outcomes of their study revealed that, when dealing with the entire Plant Village dataset, MobileNetV2 achieved an accuracy rate of 97.32%, Xception achieved 98.30%, and the ensemble model outperformed both with the highest accuracy rate of 99.10%. Notably, the accuracy of Xception and MobileNetV2 models saw improvements of 0.8% and 1.8%, respectively, when the ensemble approach was employed. Furthermore, the ensemble model showcased exceptional performance, achieving an impressive score of 99.52% across all evaluation metrics in a dataset defined by the user. In paper^20^, the researchers proposed a deep learning architecture called EfficientNet to classify tomato diseases, using a dataset of 18,161 tomato leaf images, both plain and segmented. They applied two segmentation models i.e. U-net as well as Modified U-net to segment the leaves and assessed their performance] in binary, six, and ten class classification which had groups like healthy vs. unhealthy leaves. The Modified U-net segmentation model achieved impressive results with 98.66% accuracy, 98.5% Intersection over Union (IoU), and a Dice score of 98.73% for leaf segmentation. EfficientNet-B7 outperformed in binary class with 99.95% accuracy and six-class classifications with 99.12% accuracy.

By using a publicly accessible dataset containing 54,306 images of both diseased as well as healthy plant leaves obtained under controlled settings, the researchers in paper^21^ conducted training for a deep convolutional neural network. The objective was to enable the model to identify 14 different crop species and distinguish between 26 diseases or their absence. Remarkably, our trained model achieved an impressive accuracy rate of 99.35% when evaluated on a separate test dataset, showcasing the practicality of this approach.

Besides this, the aforementioned contribution of the researchers is also presented in Table 2 to compare as well as analyse their work so that some research gaps can be traced out.

**Table 2:** Analysis of the previous work.

Refs.	Dataset	No. of images	Techniques	Outcomes	Limitation/remarks
^11^	Cauliflower image		InceptionV3	Accuracy = 90.08%	The authors stated that InceptionV3 accurately and effectively recognize diseases in cauliflower plants
			Random Search	Accuracy = 81.68%	
^2^	VegNet dataset	656 original images of cauliflower	–	–	This dataset is suitable for developing robust applications focused on the accurate classification and detection of cauliflower-related issues
^13^	–	2500 images of cauliflower	InceptionV3	Accuracy = 93.93%	The authors suggested that leveraging advanced CNN model improve the automation of plant disease detection and classification
^14^	–	776 images of cauliflower	Random forest	Accuracy = 89%	Small dataset
^15^		655 cauliflower images	2D-LeNet	Accuracy = 99%	Small dataset
^16^	Xuebai dataset	4790 images of cauliflower	MobileNetV2	Accuracy = 99.27% Loss = 0.033 F1 score = 99.24%	Limited tested dataset
^17^	–	Raw images of eggplant	DenseNet201	Accuracy = 99.06% Precision = 99.45% Specificity = 99.49% Sensitivity = 87.30%	Class imbalance
^18^	Plant Village dataset	28,310 leaf images	DenseNet, CNN	Accuracy = 98.70% Precision = 99% Recall = 99% F1 score = 99% Loss = 0.086	The parameters of CNN layers could be fine tuned to enhance the accuracy
^19^	Plant Village dataset	54,305 images of leaves	Lite ensemble MobileNetV2 and Xception	Accuracy = 99.52%	High computational time
^20^	Plant Village dataset	18,161 images tomato leaves	EfficientNet B4	Accuracy = 99.89%	The model needed to be trained with diverse dataset so that it can work for real time dataset too
^21^	PlantVillage Dataset	54,306 plant images (healthy and diseased)	Deep convolutional neural network	Accuracy = 99.35%	Limited generalization, improvement in data dependency

After assaying the table on the hand, it has been found that the study of the researchers underscores few shortcomings such as relatively small size and limited testing. The presence of class imbalance raises concerns about model performance, necessitating further exploration into fine-tuning the convolutional neural network (CNN) layers to achieve higher accuracy. Moreover, the computational demands associated with the chosen models, as well as the need for a more diverse dataset for broader real-time applicability, highlight the gaps in practical implementation. Additionally, their work also hints at limited generalization and emphasizes the importance of reducing data dependency. Therefore, the research gap centres on the need for comprehensive investigations addressing these limitations to pave the way for more robust and widely applicable cauliflower disease detection systems in agriculture.

But on the other hand, it has been also found that artificial intelligence techniques are beneficial in detecting the quality of agricultural products. From the background study, it has been found that deep learning models have shown a strong learning ability not only in the case of feature extraction but also in the classification to classify various cauliflower-based diseases. Hence based on it, we have combined the applied advanced deep learning models to develop a cauliflower disease detection model.

## Materials and methods

**Figure 2:**
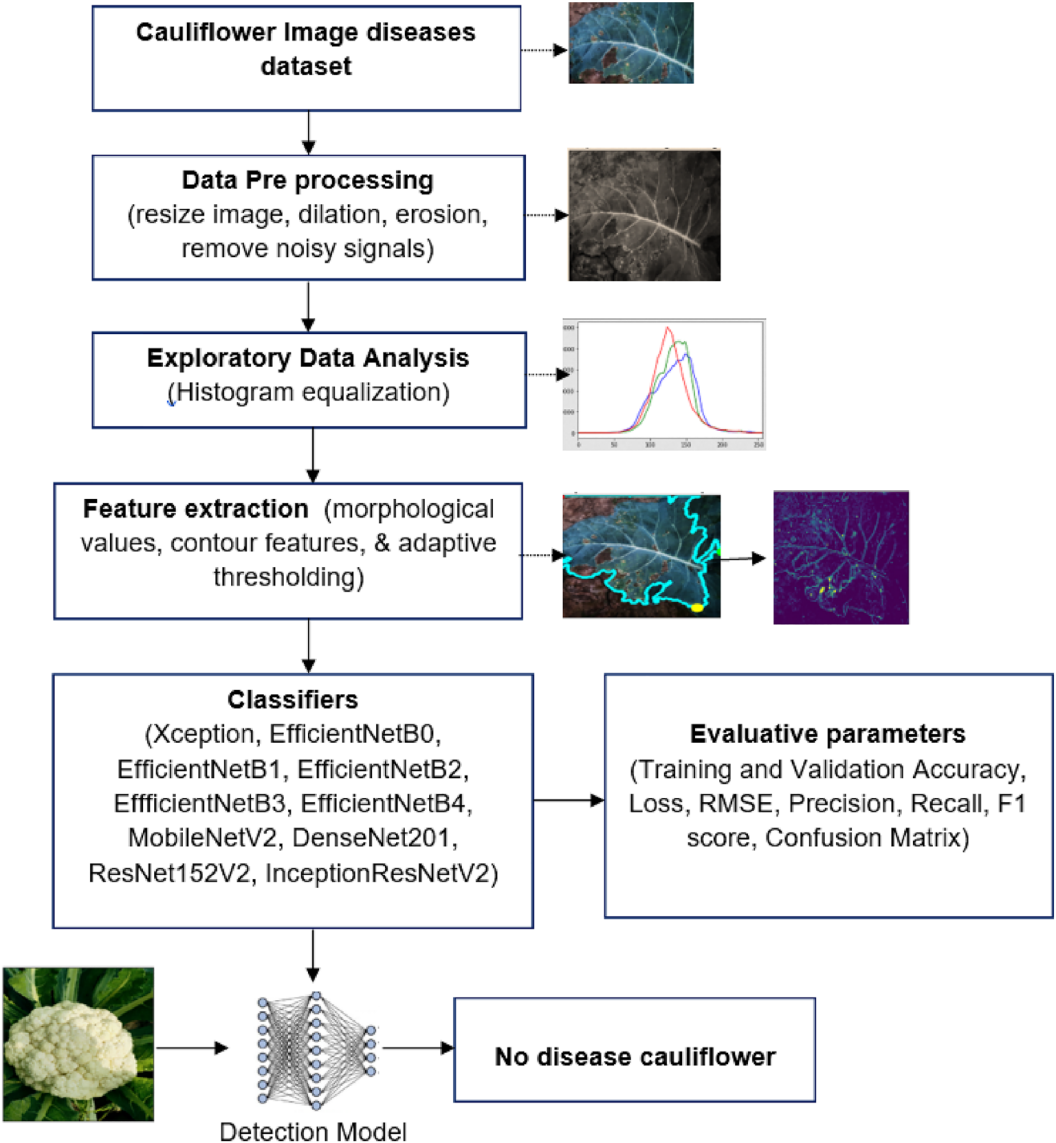
System design for detection and classification of cauliflower diseases.

The flow of the research has been mentioned and framed in this section (as shown in Fig. 2). The section holds a description of the dataset used, pre-processing techniques applied, exploratory data analysis, extracting the features, applying learning models, and parameters to predict the performance of the used models.

### Dataset used

For this research paper, two files of cauliflower images have been taken, i.e., an original and an augmented image file in which the three classes of diseases were described. In addition, the image of disease-free cauliflower has also been included in the dataset. For this dataset, the original images of six hundred fifty-six and seven thousand three hundred and sixty augmented were compiled to create the dataset. All the images of cauliflower, i.e., the disease affected and disease free, have been assembled from the Manikganj which is the vegetable production area of Bangladesh. Table 3 shows the total number of images taken from each dataset class^2^.

**Table 3:** Class-wise sample images.

Category	Total number of images
Bacterial Spot Rot	1730
Black Rot	1800
Downy Mildew	2060
No disease	1770

### Data pre-processing

**Figure 3:**
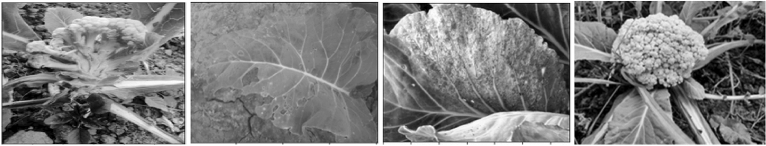
Pre-processed images of cauliflower.

The images of size 224*224 were initially imported and displayed for OpenCV (name, flag)-based data preprocessing using the window (name, flag) command. Later, the images are resized by adjusting the image's height and width to preserve the aspect ratio. As the original images have three color channels i.e. Red, Green, and Blue, so they are converted to grayscale by using the method cvtColor(), as shown in Fig. 3 to reduce the complexity of the data and simplify the architecture of computer vision models. Besides this, two morphological operations i.e. dilation as well as erosion are also applied to either add or remove the pixels from the boundaries of the image so that the from the output image, smallest value of the pixel can be obtained.

### Exploratory data analysis

**Figure 4:**
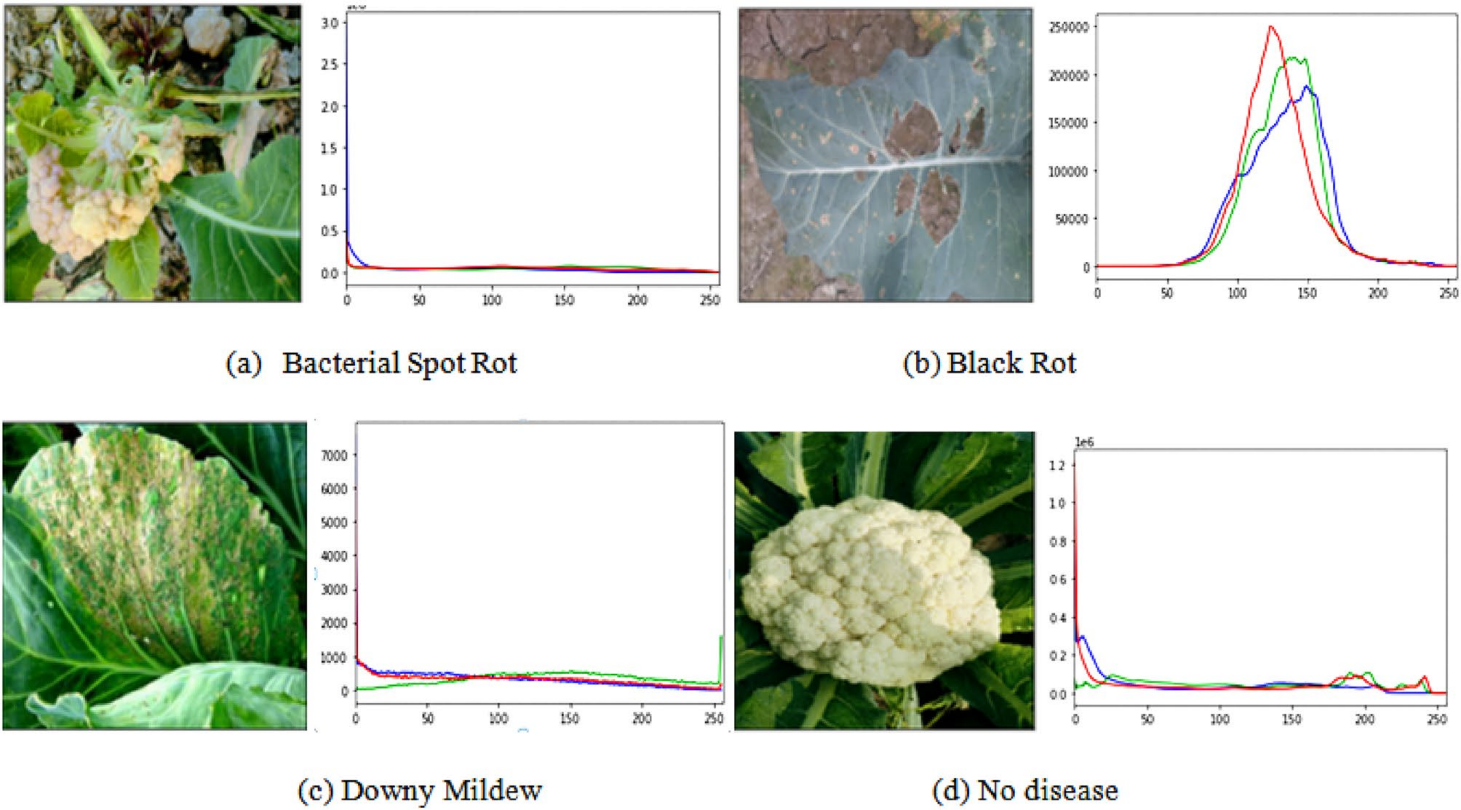
EDA of cauliflower images.

The image data has also been visually demonstrated to present the pixel intensity of the image as shown in Fig. 4. A histogram of red, green, and blue colors has been shown, which contains the quantified value of the of pixels to represent the value of their intensities.

Accordingly, Histogram Equalization (HE) is used to broaden the intensity range. In other terms, the histogram equalization method works on the distribution of those intensity values that are frequently shown, which as a result, improves the contrast in the image.

### Feature extraction

Extracting the features from the complete image is an important phase as it reduces the space as well as the time complexity of the model to process the data. Hence, in this section, the required region has been extracted by initially obtaining the properties of the images in the form of a number of parameters that include the image's area, which is the product of its height along with width as shown in Eq. (1).

1 $$area = height*width$$

where height and width defines the shape attribute and are computed using Eqs. (2) and (3)

2 $$height = cv2.boundingRect\left( {cnt} \right)$$

3 $$width = cv2.boundingRect\left( {cnt} \right)$$

In addition, we computed epsilon from Eq. (4) for determining the distance of x and y points which belong to their respective class, the aspect ratio (Eq. 5) to find the relationship between the width and height of image and the perimeter of image is obtained by using Eq. (6).

4 $$epsilon = \sqrt {\left( {x_{2} - x_{1} } \right)^{2} + \left( {y_{2} - y_{1} } \right)^{2} }$$

5 $$Aspect \;Ratio = \frac{width}{{height}}$$

6 $$Perimeter = 0.1*cv2*arclength \left( {cnt,True} \right)$$

The other parameters of the image such as extent (Eq. 7) which is the ratio of an area in an image and the rectangle that bounds the feature while as, equivalent diameter is calculated from the Eq. (8).

7 $$Extent = \frac{object \;area}{{bounding\, rectangle\; area}}$$

8 $$Equivalent \;diameter = \sqrt {\frac{4*contour\; area}{\pi }}$$

Furthermore, max, min value along with the locations is calculated using Eq. (9–12) followed by the mean color for finding the intensity values of the color using Eq. (13).

9 $$Minimum \;value = cv2.\min ()$$

10 $$Maximum \;value = cv2.\max ()$$

11 $$Minimum\; value\; Location = cv2.{\text{minMaxLo}}()$$

12 $$Maximum \;value\; Location = cv2.{\text{minMaxLo}}()$$

13 $$Mean Color = cv2.{\text{mean}}()$$

Additionally, the extreme left and rightmost points are determined, with the Eqs. (14, 15) ones being mainly accountable for computing these points, where 0 denotes the computation of quantities in the horizontal direction.

14 $$Extreme \;Leftmost\; point = tuple(cnt(cnt\left[ {:,:,0} \right].argmin()\left[ 0 \right])$$

15 $$Extreme \;Rightmost \;point = tuple(cnt(cnt\left[ {:,:,0} \right].argmin()\left[ 0 \right])$$

On the other hand, extreme bottommost and topmost points are acquired, using the Eqs. (16, 17) where 1 denotes evaluating values in the vertical direction.

16 $$Extreme \;Topmost \;point = tuple(cnt(cnt\left[ {:,:,1} \right].argmin()\left[ 0 \right])$$

17 $$Extreme\; Bottommost\; point = tuple(cnt(cnt\left[ {:,:,1} \right].argmin()\left[ 0 \right])$$

All the values for the images of four classes of dataset using these parameters have been computed and shown in Table 4.

**Table 4:** Morphological values of cauliflower images.

Perimeter	Bacterial spot rot	Black rot	Downy Mildew	No disease
Area	2.0	1.0	7.0	1.0
Perimeter	2.0	0.0	12.0	0.0
Epsilon	0.2	0.0	1.20	0.0
Width	2	1	7	1
Height	1	1	1	1
Equivalent diameter	0.2	0.3	0.2	0.4
Extent	1.0	2.0	2.0	1.0
Aspect ratio	2.0	1.0	7.0	1.0
Max value	145.0	128.0	132.0	130.0
Min value	131.0	128.0	128.0	130.0
Min value loc	(3619,2999)	3822,3023	63,249	722,3999
Max value loc	(3618,2999)	3822,3023	64,249	722,3999
Mean color	(138.0)	128.0	129.28	130.0
Extreme Leftmost point	(3628,2999)	3822,3023	63,249	722,3999
Extreme rightmost point	(3619,2999)	3822,3023	69,249	722,3999
Extreme topmost point	(3618,2999)	3822,3023	63,249	722,3999
Extreme bottommost point	(3618,2999)	3822,3023	63,249	722,3999

After obtaining the different values of the images, continuous curves were generated to obtain the extreme points and the largest contour based on which the image had been cropped. The cropped image has been later sent for adaptive thresholding, where the neighboring pixels are considered at a time to compute the threshold value for any specific region for performing segmentation. Well, in adaptive thresholding, we already know that for each smaller region, a threshold value is calculated, and as there are so many regions, there are various threshold values.

**Figure 5:**
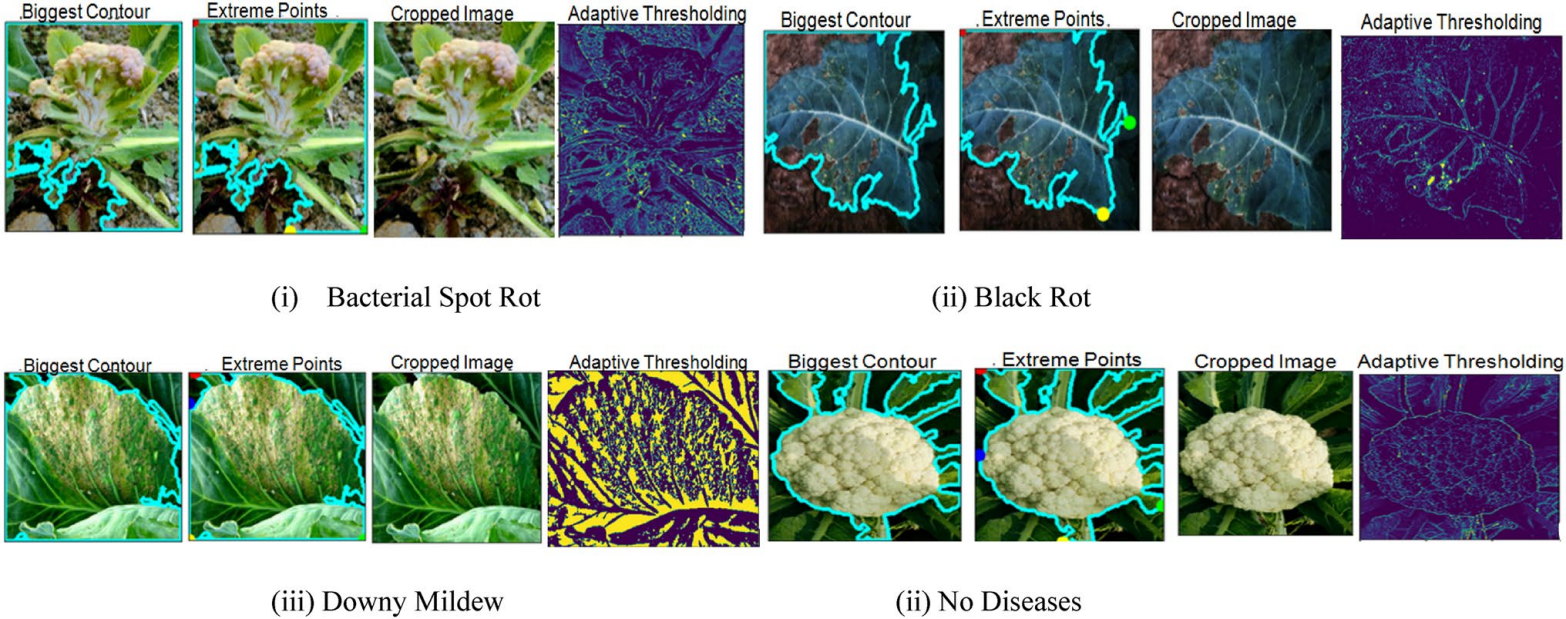
Feature extraction techniques on cauliflower images.

For this research, we have used OpenCV () to perform an adaptive threshold operation in which cv2.adaptiveThreshold() has been used. Five parameters have been passed, i.e., an array of the input image, assignment of maximum value to the pixel, type of adaptive thresholding, size of neighborhood pixels for calculating the threshold value, and a constant value subtracted from the mean of the neighborhood pixels (in Fig. 5).

After features were extracted, the data was divided into training and validation sets with 3:1 ratio utilizing 1384, 1440, 1648, and 1416 training images from the datasets for bacterial spot rot, black rot, downy mildew, and no disease. On the contrary, 346, 360, 412, and 354 validation images were taken from the same dataset, as mentioned earlier.

### Applied models

After the images from the dataset are enhanced efficiently, they are fed to the pre-trained models for further computation, such as ResNet152V2, Xception, EfficientNetB1, EfficientNetB0, EfficientNetB3, EfficientNetB2, EfficientNetB4, DenseNet201, and InceptionResNetV2.

**Figure 6:**
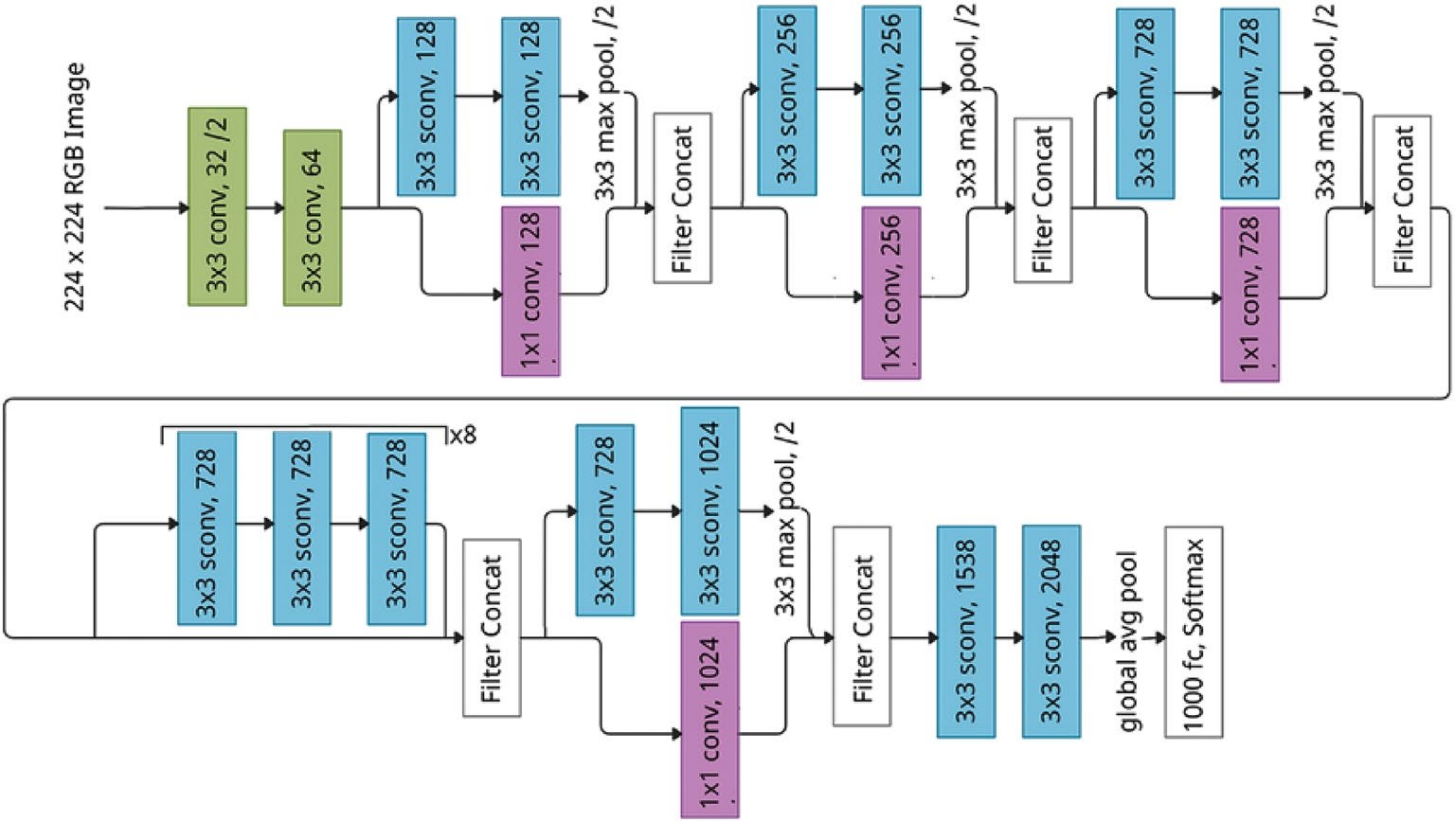
Architecture of Xception model.

**Xception**: The Xception model is the 71-layer deep CNN model in which the layer slices the output into three segments and then sends it to the next set of filters. The 1*1 filter is for the single convolutional level, while as 3*3 filter is for the three convolutional layers. It has also been studied that, unlike the CNN model, the Xception model involves both point-wise and depth-wise convolution. In this research work, 20,861,480 parameters of Xception with output shape (None, 7,7,2048) have been used. Besides this, the crux of the architecture has globalaveragepooling2d (None, 2048) with 0 parameters, dense layer (None, 256) with 524,288 parameters, batch normalization (None, 256) with 1024 parameters, activation function and dropout (None, 256) with 0 parameters, and at the end second dense layer (None,2) with 1285 parameters. Figure 6 shows the block view and dataflow of Xception model^22^.

**Figure 7:**
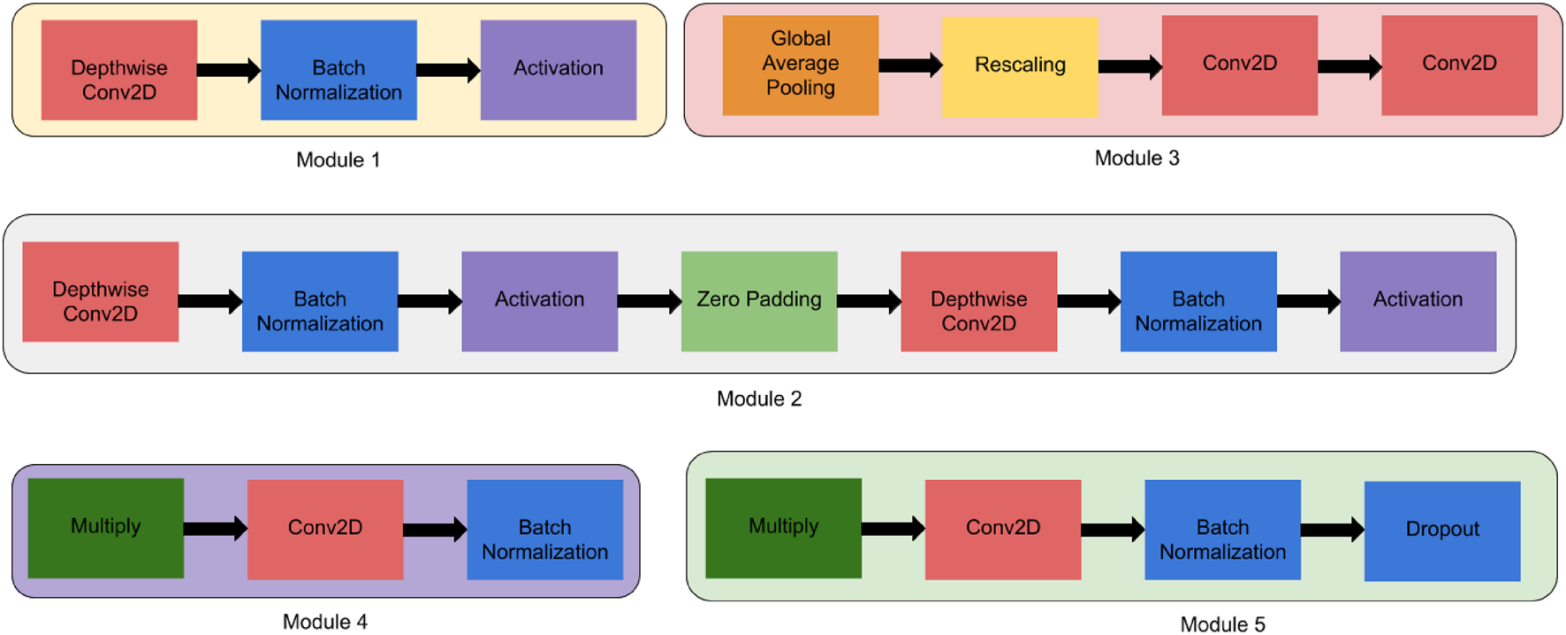
Modules used in the architecture of all versions of EfficientNet model.

**EfficientNet models**: In this research work, various versions of EfficientNet models have been used such as EfficientNetB0, EfficientNetB1, EfficientNetB2, and EfficientNetB3. EfficientNet neural network is basically a new scaled up baseline network that use AutoML MNAS framework to optimize the efficiency and accuracy. The model consists of various blocks such as input layer, rescaling, normalization, zero padding, Conv2D, Batch Normalization, and Activation as shown in Fig. 7^23^.

**Figure 8:**
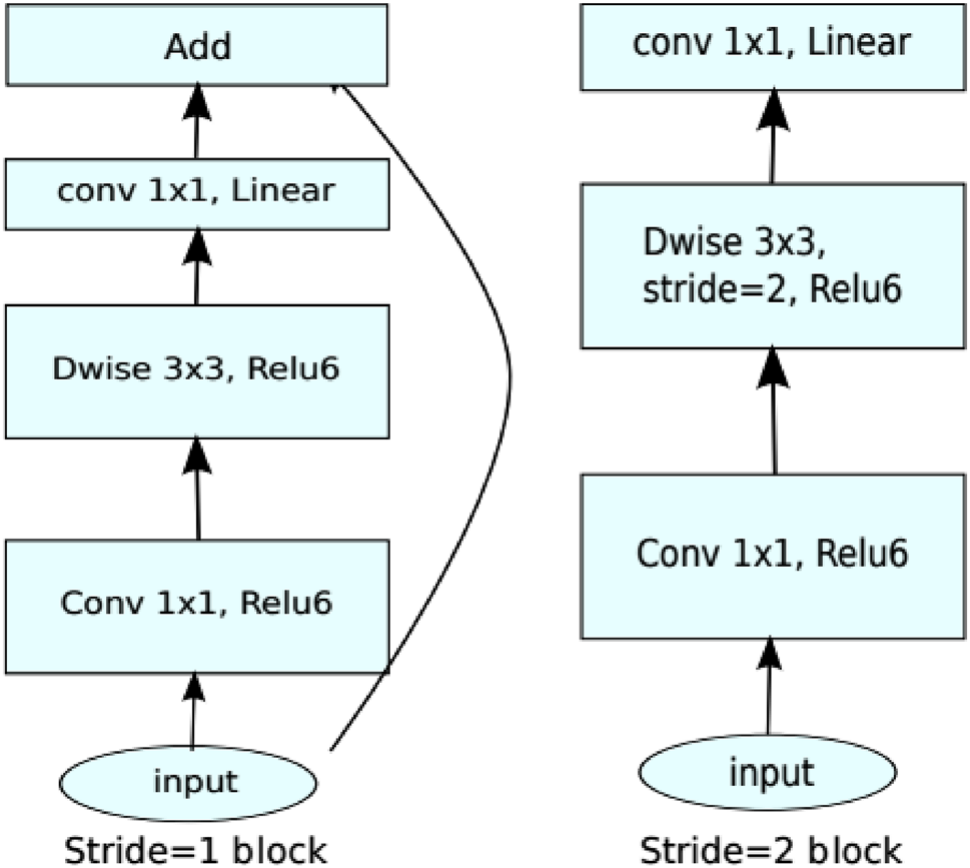
Architecture of MobileNetV2.

**MobileNetV2**: This type of model has 53 convolution layers and 1 average pool layer. It consists of two main components i.e., inverted residual block and bottle residual block as shown in Fig. 8. In addition to this, mobilenetv2 architecture also has two types of convolution layers i.e., 1 × 1 convolution and 3 × 3 depth wise convolution. Besides it, each block of it has 3 types of different layers i.e., 1 × 1 convolution layer with Relu6 activation function, depth wise convolution, and 1 × 1 convolution with non-linear layer^24^. In this research work, 2,257,984 parameters of MobileNetV2 with output shape (None, 7,7,1280) has been used in which global average pooling and dropout (None, 1280) with 0 parameter and dense layer (None, 6) with 7686 parameter is being used.

**Figure 9:**
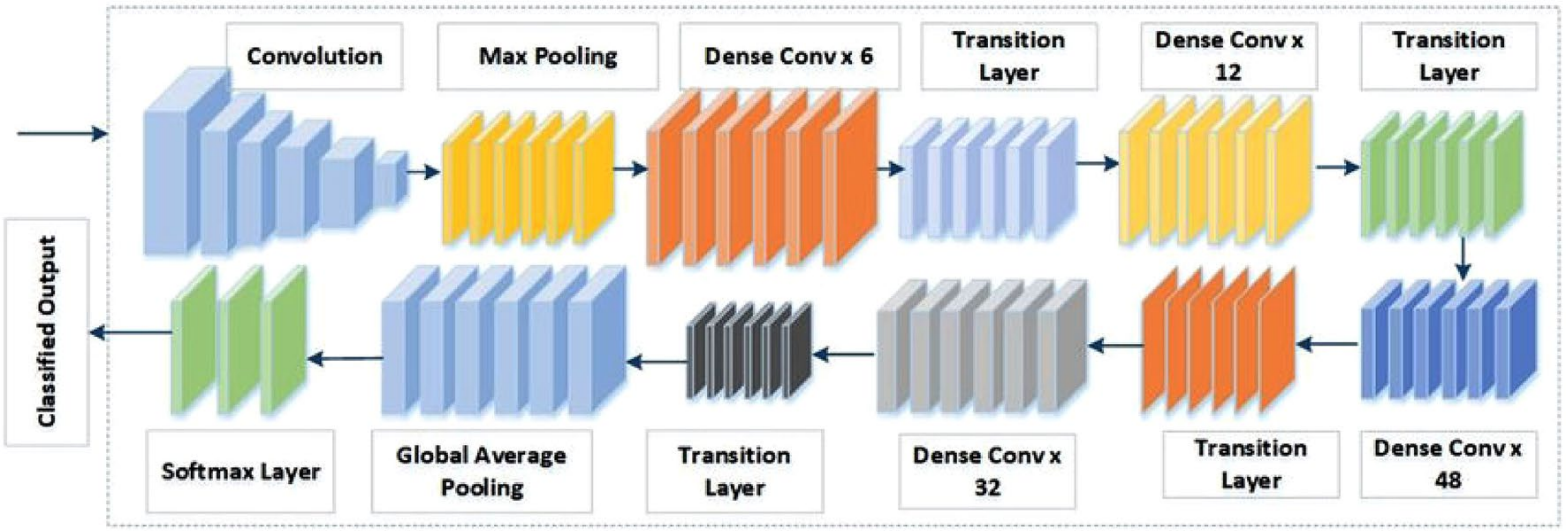
Architecture of DenseNet201.

**DenseNet201**: DenseNet201 is a CNN model which is utilized to bring out features by learning weights of the input from the ImageNet dataset. The DenseNet201 has shown the best performance on various datasets because of having direct connections from all preceding layers to all subsequent layers which are shown in figure. In this research work, we have added two dense layers for classification having 128 and 64 neurons respectively. The feature extraction network i.e. DenseNet201 followed by softmax activation function for multi class—classification as shown in Fig. 9^25^. The total parameters of DenseNet201 used are 18,815,813 out of which 18,586,245 and 229,568 are trainable and non-trainable parameters, respectively. Other layers that have been used are globalaveragepooling2d (None, 1920) with 0 parameter, dense layer, batch normalization, activation function, dropout (None, 256) with parameters 0,491520,1024,0,0 respectively. At the end the dense layer (None, 5) with parameter 1285 is being used for the classification of the input.

**Figure 10:**
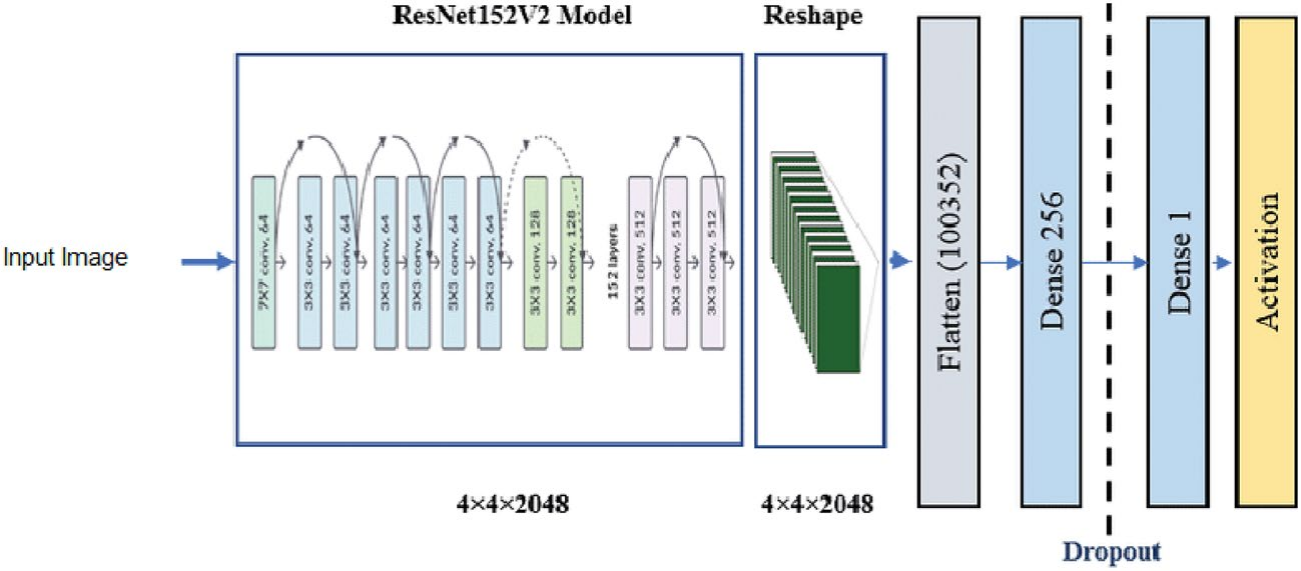
Architecture of ResNet152V2.

**ResNet152V2**: Residual network is a convolutional neural network which comprise of thousands of convolution layers. V2 uses batch normalisation before each weight layer, whereas the previous version of ResNetV1, did not. ResNet's impressive performance in image recognition and localization tasks demonstrates the significance of many visual recognition tasks. In this research work, 152 layers of residual network have been used for classification which has reshape step, first dense layer, flatten step, second dense layer, a dropout layer, and finally an activation function (not in sequential form) for classifying the image as shown in Fig. 10^26^. ResNet152V2 has 58,858,245 parameters, with 58,713,989 trainable parameters and 144,256 non-trainable parameters. Besides this, the other layers that have been used in the architecture are globalaveragepooling2d (None, 2048) with 0 parameters, dense layer, batch normalization, activation function, and dropout having output shape (None, 256) each with 524288, 1024, 0, 0 respectively. At the end, dense layer is being used with output shape (None, 5) and 1285 parameter value.

**Figure 11:**
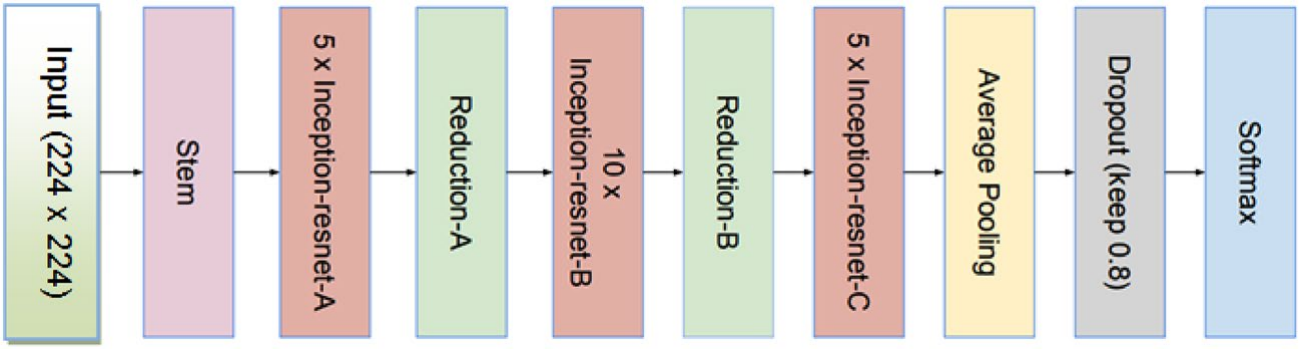
Architecture of InceptionResNetV2.

**InceptionResNetV2**: It is a hybridization of ResNetV2 and Inception model in order to preserve the various characteristics of the multi-convolutional network and improve the model's classification accuracy. This enhanced version of the Inception architecture expedited the model and dramatically enhanced performance. The InceptionResNetV2’s architecture is demonstrated in Fig. 11^27^. InceptionResNetV2 has a total parameter count of 54,732,261: out of which 54,671,205 are for trainable parameters and 61,056 are for non-trainable parameters. This architecture has globalaveragepooling2d with output shape (None, 1536) with 0 weights, dense layer, batch normalization, activation function, and dropout with output shape (None, 256) and 393216, 1024, 0, 0.8 parameters. At the end, the architecture has second dense layer of output shape (None, 5) with 1285 parameters.

### Evaluative parameters

Various evaluation parameters have been employed to evaluate all applied models. Accuracy is one of these measures, measuring the degree of precision attained by the model during training and validation on the dataset. The metrics also include loss, which reflects how well the model has been trained and verified and is the opposite of accuracy. In this assessment method, the root mean square error is also used as a statistic.

Similarly, we have evaluated the performance of models using precision, F1 score, and recall. Precision refers to how well the model predicts the class, whereas recall refers to the number of times the model correctly defines the relevant class. The F1 score, in this instance, indicates the average of recall and accuracy. The F1 score essentially acts as an indicator of a model's precision when used on a certain dataset. Table 5 provides the formulae that the models used to determine these parameters^28, 29^.

**Table 5:** Formulae to compute the performance metrics of models.

Evaluative parameters	Formula
Accuracy	$\frac{True\; Positive + True \;Negative}{{True \;Positive + True \;Negative + False \;Positive + False \;Negative}}$
Loss	$\frac{{\left( {Actual\; Value - Predicted\; Value} \right)^{2} }}{Number\; of \;observations}$
RMSE	$\sqrt {\frac{{\left( {Actual\, Value - Predicted\, Value} \right)^{2} }}{Number\; of \;observations}}$
Precision	$\frac{True\, Positive}{{True Positive + False\, Positive}}$
Recall	$\frac{True\, positive}{{True\, positive + False\, Negative}}$
F1 score	$2\frac{Precision*Recall}{{Recall + Precision}}$

## Analyzing the results

The execution of the models such as Xception, EfficientNet B0, EfficientNetB1, EfficientNetB2, EfficientNetB3, EfficientNetB4, MobileNetV2, DenseNet201, ResNet152V2, and InceptionResNetV2 that have been used to detect and classify various diseases of cauliflower have been evaluated using the performance evaluation parameters. These models' layers were hyper-tuned to enhance the classification accuracy. The learning rate taken was 0.0001, the sigmoidal activation function, and the dropout was 0.5 was used to perform multi-class. Initially, the models were computed during the training and validation phases for the combined dataset, and later, the confusion matrix was generated to evaluate their performances for different classes such as that have been taken.

Table 6 shows that EfficientNetB1 obtained the highest accuracy value of 99.37%, Xception got the best loss, and root mean square error value by 0.19 and 0.44, respectively. Likewise, during the validation phase, the best accuracy has been again obtained by EfficientNetB1 by 99.90%, along with the loss and root mean error square value by 0.16 and 0.40, respectively. On the contrary, the model that computed the least accuracy among all is ResNet152V2 by 52.42% and the worst loss as well as rmse value by 5.92 and 2.43, respectively.

**Table 6:** Performance of models.

Models	Training			Validation		
	Accuracy	Loss	RMSE	Accuracy	Loss	RMSE
Xception	99.25	**0.1991**	**0.446206**	90.91	0.4594	0.677791
EfficientNetB0	99.25	0.2139	0.462493	98.48	0.2139	0.462493
EfficientNetB1	**99.37**	0.2134	0.461952	**99.90**	**0.1606**	**0.400749**
EfficientNetB2	98.50	0.2918	0.540185	98.48	0.2535	0.503488
EfficientNetB3	94.25	0.5200	0.72111	96.97	0.5547	0.744782
EfficientNetB4	97.87	0.2799	0.529056	92.42	0.3701	0.608358
MobileNetV2	95.00	0.4428	0.665432	66.06	1.5677	1.252078
DenseNet201	98.25	0.2536	0.503587	98.48	0.3244	0.569561
ResNet152V2	66.50	0.9321	0.965453	52.42	5.9270	2.434543
InceptionResNetV2	97.62	0.2475	0.497494	95.45	0.3382	0.58155

Bold denotes the best results for each parameter out of all results.

**Figure 12:**
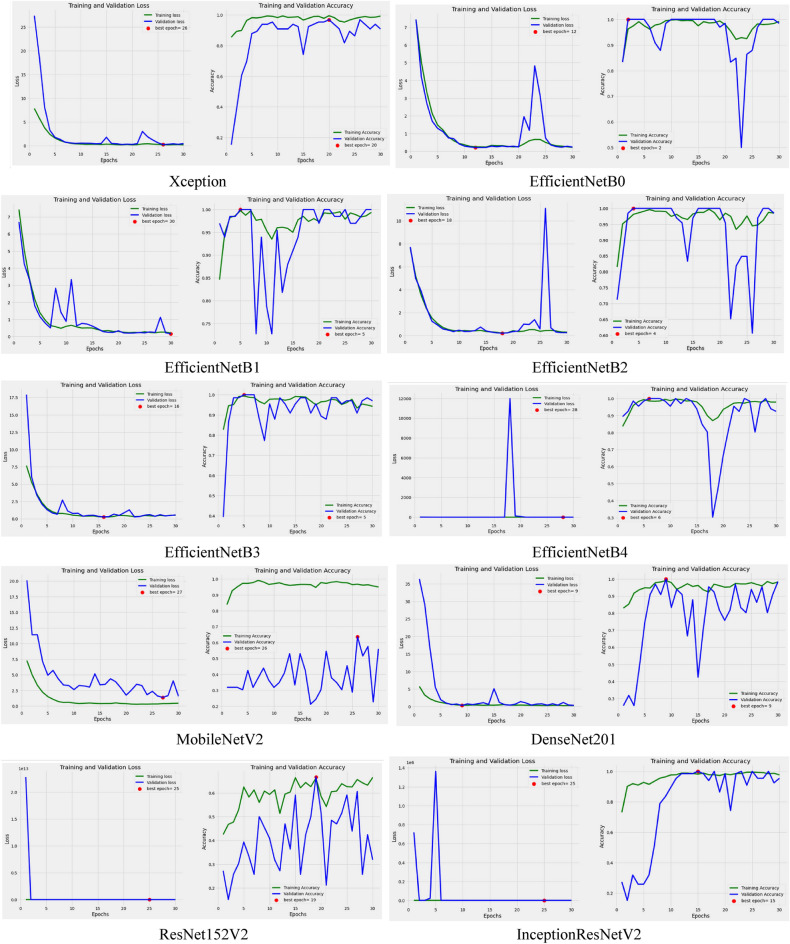
Graphical analysis of models for detection of cauliflower diseases.

The graphical analysis of the models has also been presented in Fig. 12 to study the pattern of their curves for training and validation accuracy as well as loss. All the models have been trained and tested for 30 epochs, out of which the best epoch has been located at which the model obtained the highest value.

On studying the curves of all the models, it has been observed that they have shown some noisy signals for both cases except a few, such as training and validation loss of ResNet152V2 and Inception ResNetV2. It has also been found that the Xception model, along with all the versions of the EfficientNet model, showed the best line of a curve at various points of epochs. Hence from the curve nature of models, it can be analyzed that the models do not show any modeling error such as overfitting and underfitting except MobileNetV2.

Based on these results, we have also tested the performance of the models based on specific parameters shown in Table 7. It has been found that Xception generated the highest precision, recall, and F1 score value by 99.65%, 99.8%, and 99.62% as compared to the other models. On the contrary, the least obtained by MobileNetV2 was by 61.81% precision score, 63.61% recall, and 68.45% F1 score.

**Table 7:** Analysis of model for other parameters.

Models	Precision	Recall	F1Score
Xception	**99.65**	**99.8**	**99.62**
EfficientNetB0	98.6	97.42	97.95
EfficientNetB1	97.42	94.92	95.85
EfficientNetB2	98.61	97.35	97.88
EfficientNetB3	98.61	97.42	97.94
EfficientNetB4	98.57	94.92	98.25
MobileNetV2	61.81	63.61	68.45
DenseNet201	99.9	99.9	99.9
ResNet152V2	70.47	73.16	63.39
InceptionResNetV2	98.61	97.42	97.96

Bold denotes the best results for each parameter out of all results.

**Figure 13:**
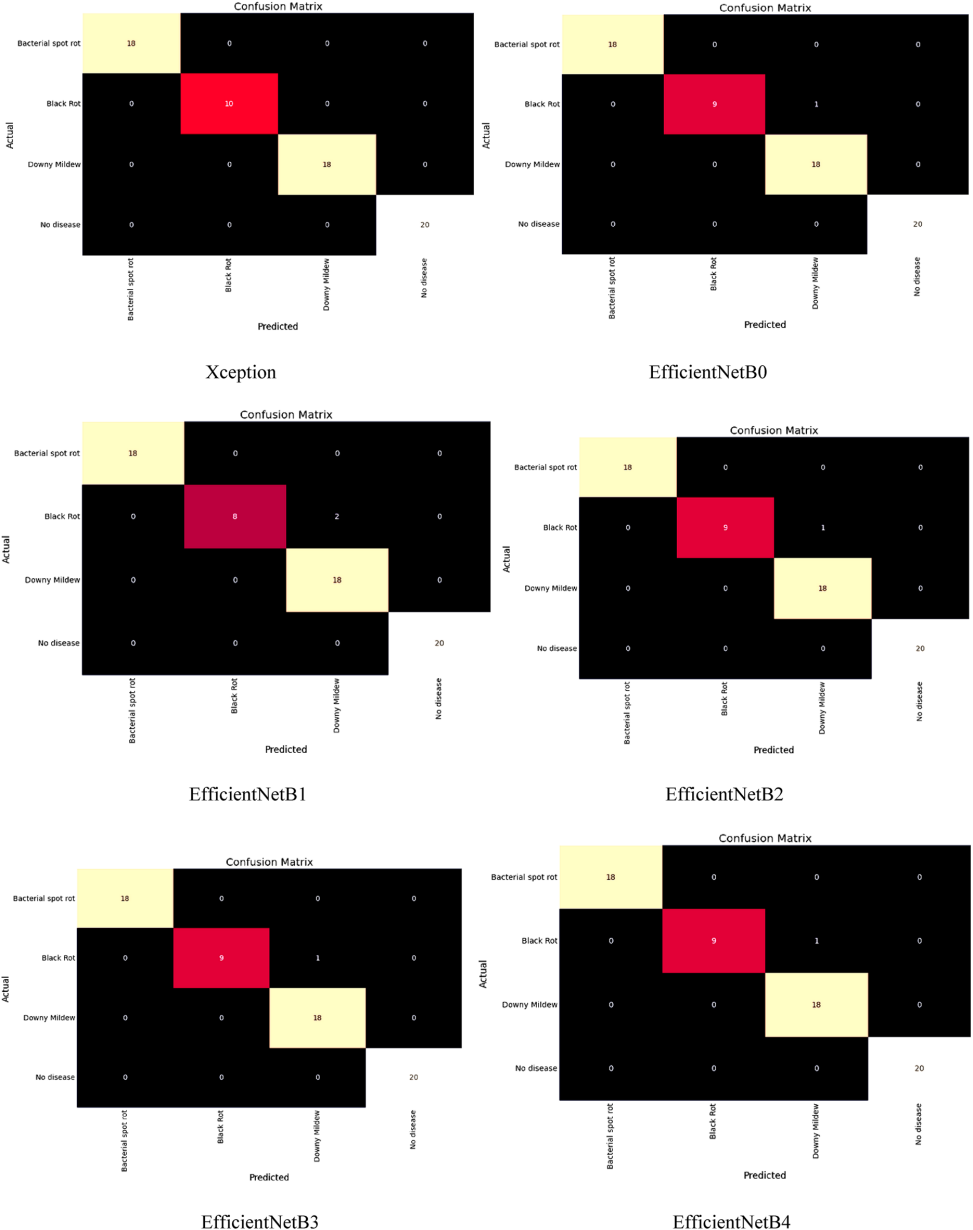

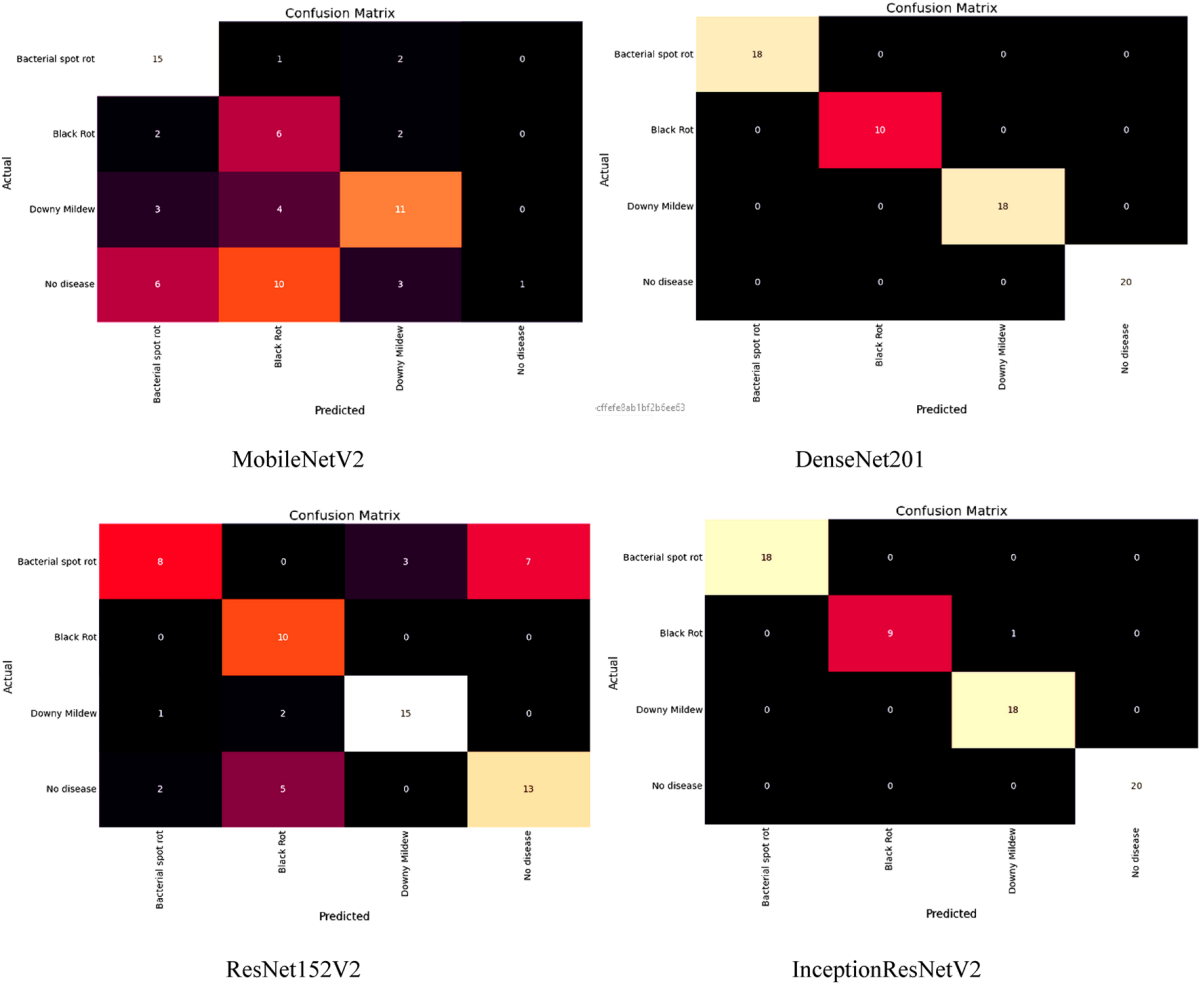
Confusion Matrix of models for detection of cauliflower diseases.

In addition, we have also evaluated models’ performances for various classes of this dataset, such as bacterial spot rot, black rot, downy mildew, and no disease. A confusion matrix of all the applied models has been generated for all the classes, such as bacterial spot rot, black rot, downy mildew, and no disease. The confusion matrix identifies which component of the classification model makes errors when making predictions, allowing us to identify both the classification model’s errors and, more importantly, the types of errors that occurred as shown in Fig. 13.

The diagonal values of this confusion matrix represent the true positive values, and the summation of horizontal values of any particular class represents the false negative. Likewise, the summation of vertical values of any specific class represents the false positive, and from the rest of the values, we compute the true negative output of the class. Using these values, we computed the accuracy, loss, rmse, recall, precision, and F1-score values of models for different classes, as shown in Table 8.

**Table 8:** Analysing models for different classes.

Class	$\mathrm{Models}$	$\mathrm{Training}$			$\mathrm{Validation}$		
		Acc	$\mathrm{Loss}$	$\mathrm{RMSE}$	Acc	$\mathrm{Loss}$	$\mathrm{RMSE}$
Bacterial Rot	Xception	**99.25**	0.1991	0.446206	96.59	0.4453	0.667308
	EfficientNetB0	95.24	0.2139	0.462493	94.26	0.2473	0.497293
	EfficientNetB1	94.36	0.4551	0.674611	96.59	**0.1592**	**0.398999**
	EfficientNetB2	98.56	0.2155	0.46422	91.48	0.2426	0.492544
	EfficientNetB3	96.59	0.4551	0.674611	95.26	0.5486	0.740675
	EfficientNetB4	92.46	0.2155	0.46422	95.46	0.3265	0.571402
	MobileNetV2	66.59	**0.1604**	**0.4005**	69.58	1.5485	1.244387
	DenseNet201	98.79	0.2546	0.504579	94.41	0.3259	0.570877
	ResNet152V2	52.56	0.5547	0.744782	53.79	5.9426	2.437745
	InceptionResNetV2	94.66	0.4551	0.674611	**99.00**	0.3782	0.61498
Black Rot	Xception	**99.59**	0.1959	0.442606	90.26	0.4551	0.674611
	EfficientNetB0	95.46	0.2146	0.463249	98.46	0.2155	0.46422
	EfficientNetB1	94.59	0.4526	0.672756	**99.59**	**0.1604**	**0.4005**
	EfficientNetB2	96.46	0.2146	0.463249	98.56	0.2546	0.504579
	EfficientNetB3	94.18	**0.1659**	**0.407308**	96.59	0.5547	0.744782
	EfficientNetB4	96.46	0.2546	0.504579	92.46	0.3789	0.615549
	MobileNetV2	94.59	0.5526	0.743371	66.59	1.5679	1.252158
	DenseNet201	95.49	0.2559	0.505866	98.79	0.3254	0.570438
	ResNet152V2	66.59	0.9379	0.968452	52.56	5.9276	2.434666
	InceptionResNetV2	94.46	0.2456	0.49558	95.59	0.3348	0.578619
Downy Mildew	Xception	95.25	0.5991	0.774016	90.59	0.4559	0.675204
	EfficientNetB0	95.25	0.2639	0.513712	98.26	0.2159	0.46465
	EfficientNetB1	95.59	0.4634	0.680735	**99.59**	**0.1606**	**0.400749**
	EfficientNetB2	**99.46**	0.5918	0.769285	98.48	0.2546	0.504579
	EfficientNetB3	97.59	0.5200	0.72111	96.26	0.5547	0.744782
	EfficientNetB4	93.13	0.2799	0.529056	92.46	0.3786	0.615305
	MobileNetV2	98.49	0.4596	0.677938	66.58	1.5677	1.252078
	DenseNet201	93.46	**0.2536**	**0.503587**	98.41	0.3259	0.570877
	ResNet152V2	69.52	0.9467	0.972985	52.79	5.9270	2.434543
	InceptionResNetV2	94.59	0.2596	0.50951	95.00	0.3346	0.578446
No Disease	Xception	**99.25**	**0.1991**	**0.446206**	91.95	0.4594	0.677791
	EfficientNetB0	95.24	0.2139	0.462493	97.46	0.2599	0.509804
	EfficientNetB1	94.36	0.4551	0.674611	98.94	0.1596	0.3995
	EfficientNetB2	96.52	0.2155	0.46422	**99.41**	**0.2465**	**0.496488**
	EfficientNetB3	94.25	0.1604	0.4005	97.93	0.5267	0.725741
	EfficientNetB4	96.89	0.2546	0.504579	91.42	0.3251	0.570175
	MobileNetV2	94.04	0.5547	0.744782	65.04	1.5467	1.243664
	DenseNet201	95.25	0.2536	0.503587	97.41	0.3494	0.591101
	ResNet152V2	66.55	0.9321	0.965453	51.42	5.9590	2.441106
	InceptionResNetV2	94.66	0.2475	0.497494	98.46	0.3562	0.596825

Bold denotes the best results for each parameter out of all results.

From the table, it has been found that during the training phase, for the bacterial spot rot class, Xception obtained the highest accuracy value of 99.25%, and MobileNetV2 computed the best loss and rmse value by 0.16 and 0.40, respectively. In the same way, for the Black Rot class, Xception once again obtained the highest accuracy by 99.59%, while the best loss and rmse of 0.16 and 0.40, respectively, were obtained by EfficientNetB3. Likewise, for Downy Mildew, EfficientNetB2 obtained the highest accuracy by 99.46%, DenseNet201 obtained the best loss, and rmse value by 0.25 and 0.53, respectively. At the end for the no disease class, Xception computed the highest accuracy, loss, and root mean square error value by 99.25%, 0.19, and 0.44, respectively.

During the bacterial spot rot class validation phase, InceptionResNetV2 obtained the highest accuracy value by 99%, and EfficientNetB1 computed the best loss and rmse value by 0.15 and 0.39, respectively. In the same way, for the Black Rot class, EfficientNetB1 obtained the highest accuracy, loss, and root mean square error by 99.59%, 0.16, and 0.40, respectively. Likewise, Downy Mildew EfficientNetB1 obtained the best accuracy, loss, and root mean square error value by 99.59%, 0.16, and 0.40, respectively. At the end for the no disease class, EfficientNetB2 computed the highest root mean square error, loss, and accuracy value by 0.49, 0.24, 99.41% respectively.

**Figure 14:**
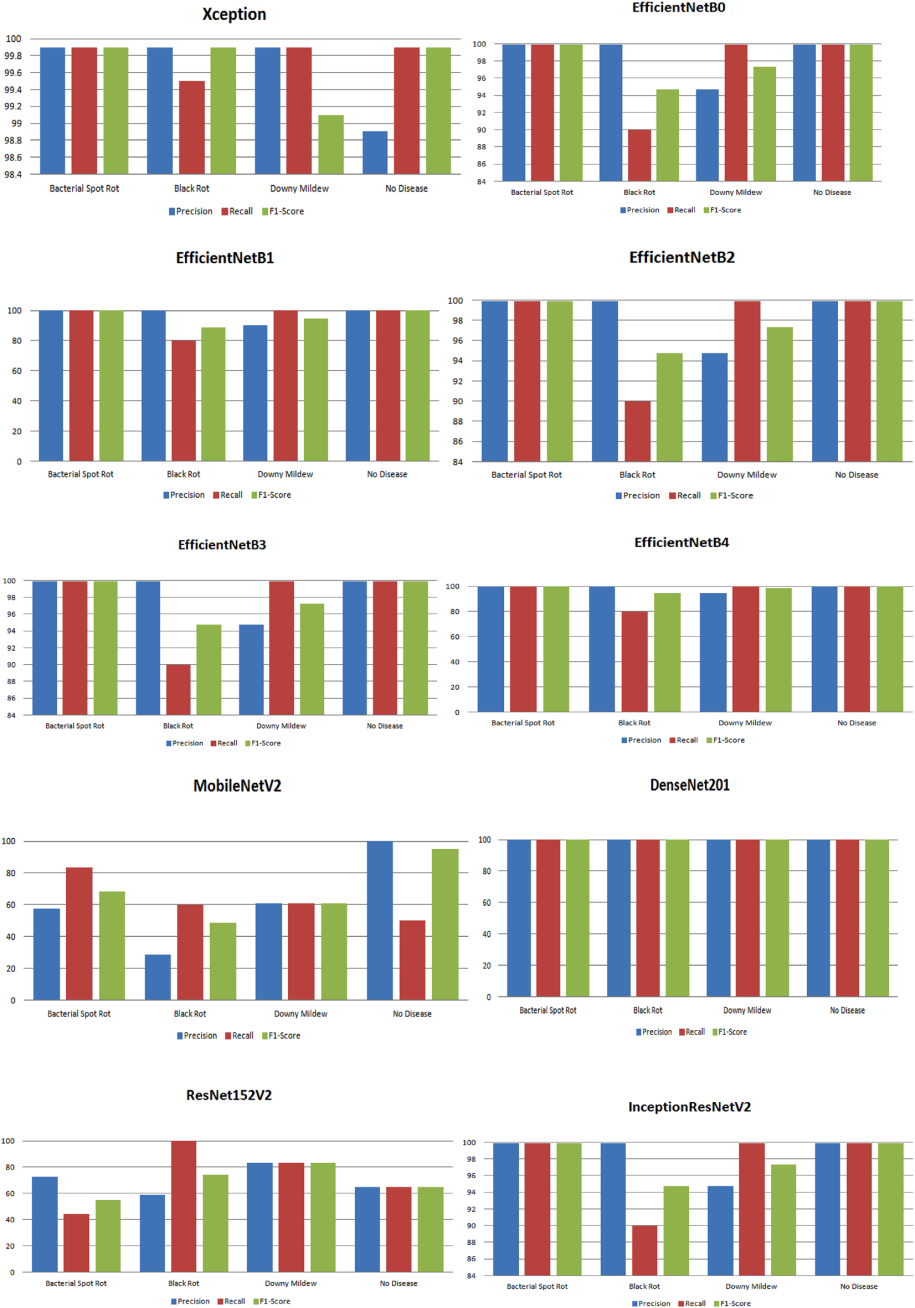
Performance evaluation of models for the classification of various diseases in cauliflower.

In addition, the recall, precision, and F1 score value of models for different classes have also been computed in Fig. 14. The Xception model obtained the best precision value of 99.9% for all the classes except no disease. Similarly, the highest recall and F1 score of 99.9% has been obtained for every class except black rot and downy mildew, respectively. All four versions of EfficientNet models, i.e., EfficientNetB0, EfficientNetB1, EfficientNetB2, and EfficientNetB3, obtained the highest F1 score, recall, and precision value by 99.9% for bacterial rot and no disease classes. The lowest value of precision, recall, and MobileNetV2 obtained an F1 score of 28.57%, 50%, and 48.71% for black rot and no disease, respectively. DenseNet201 showed the best performance among all the models as this model computed the highest recall, precision, and F1 score of 99.9% for all the classes. ResNet152V2 obtained the highest precision and F1 score for class Downy Mildew by 83.33% and the highest recall of 99.9% for black rot. InceptionResNetV2 obtained the highest precision value of 99.9% for all the classes except downy mildew. Similarly, the highest recall of 99.9% has been obtained by all the classes except Black rot. In the end, the best F1 score of 99.9% has been obtained for the classes such as bacterial spot rot and no disease class.

Besides this, the computational time of the applied models have been also computed and mentioned in Table 9. It has been found that ResNet152V2 took the maximum time i.e. 1 h 36 min 2 s while as the least has been computed by EfficientNetB0 i.e. 24 min 49 s.

**Table 9:** Computational time of models.

Models	Time
Xception	28 min 6 s
EfficientNetB0	24 min 49 s
EfficientNetB1	30 min 52 s
EfficientNetB2	48 min 6 s
EfficientNetB3	35 min 89 s
EfficientNetB4	60 min 44 s
MobileNetV2	20 min 94 s
DenseNet201	40 min 28 s
ResNet152V2	1 h 36 min 2 s
InceptionResNetV2	45 min 8 s

In addition, we have also compared the accuracy of our proposed system of this research work with the accuracies obtained by the existing techniques in Table 10. Our research work has been found to perform better to identify and classify cauliflower diseases, as out of the ten applied models, EfficientNetB1 obtained the top accuracy of 99.90%. InceptionV3 calculated the lowest accuracy value in the deep neural network by 93.93%. On the contrary, while comparing the results of a machine and deep learning over the best model i.e. EfficientNetB1, the best one has been taken by deep neural networks only.

**Table 10:** Proposed work comparison with the existing work.

Refs.	Dataset	Techniques	Accuracy (%)
^11^	Sample images of cauliflower taken from Bangladesh	Random forest	81.68
^13^	2500 images of cauliflower	Inception V3	93.93
^14^	776 images of cauliflower	Random Forest	89
^16^	Images of Xuebai	MobileNetV1	95.63
^17^	Images of Eggplant diseases	DenseNet201	99.06
Our Study	VegNet	EfficientNetB1	99.90

## Conclusion

Human health depends on vegetables which is an important part of agriculture. Information technology helps vegetable producers to increase yields, promote global food security and sustainable agriculture. To prevent people from becoming ill, we have developed a cauliflower disease detection system as part of this research. To summarise the work, 7360 images from four distinct classes which includes bacterial spot rot, downy mildew, black rot, and no disease, had been used for the training of models such as. Here, Gaussian, Erosion, and Dilation techniques were used to pre-process the images before analyzing the RGB pixel intensities. The contour feature extraction and adaptive thresholding techniques were used to generate morphologic values and cropped images. Ten models were subsequently trained and validated using the aforementioned four-class dataset where EfficientNetB1 obtained the highest validation accuracy of 99.90%, loss of 0.16, as well as root mean square error of 0.40. In addition, there are few limitations, such as the bacterial spot rot class, which contains the image of a dataset with no diseases and can lead to the misbalance or misclassification of other input classes. Additionally, the contour feature extraction failed on a few images, which can also lead to incorrect classification and detection of the input image. Consequently, in the future, the contour feature extraction on the images of the VegNet dataset should be enhanced, and a vegetable disease detection system should be proposed that detects not only cauliflower but also diseases on other vegetables.

Overall, this work signifies a crucial step towards harnessing the power of information technology in agriculture. By providing accurate and efficient cauliflower disease detection, we contribute to increased yields, global food security, and sustainable farming practices. As we continue to refine and expand our methods, we aim to make a lasting impact on agriculture, ultimately benefitting both farmers and consumers worldwide.

## Data Availability

The data that support the findings of this study is publically available on the given link: https://data.mendeley.com/datasets/t5sssfgn2v/3 (Accessed Date: 13th September, 2023).
