# Author Correction: Advanced deep learning techniques for early disease prediction in cauliflower plants

**DOI:** 10.1038/s41598-023-47732-2

**Published:** 2023-11-24

**Authors:** G. Prabu Kanna, S. J. K. Jagadeesh Kumar, Yogesh Kumar, Ankur Changela, Marcin Woźniak, Jana Shafi, Muhammad Fazal Ijaz

**Affiliations:** 1 School of Computer Science and Engineering, VIT Bhopal University, Bhopal-Indore Highway, Kothrikalan, Sehore Madhya Pradesh - 466114, India; 2 Department of Computer Science and Engineering, Kathir College of Engineering, Neelambur, India; 3 Department of CSE, School of Technology, Pandit Deendayal Energy University, Gandhinagar, Gujarat India; 4 Faculty of Applied Mathematics, Silesian University of Technology, 44-100 Gliwice, Poland; 5 Department of Computer Science, College of Arts and Science, Prince Sattam bin Abdul Aziz University, 11991 Wadi Ad-Dawasir, Saudi Arabia; 6 School of IT and Engineering, Melbourne Institute of Technology, Melbourne, 3000 Australia; 7 Department of ICT, School of Technology, Pandit Deendayal Energy University, Gandhinagar, Gujarat, India

Correction to: *Scientific Reports* https://doi.org/10.1038/s41598-023-45403-w, published online 27 October 2023

The Acknowledgements section in the original version of this Article was omitted. The Acknowledgements section now reads:

“This study is supported via funding from Prince Sattam bin Abdulaziz University project number (PSAU/2023/R/1445).”

The original Article has been corrected.

